# Protective effect and mechanism of SIRT1 under stress-induced vascular senescence

**DOI:** 10.1515/biol-2025-1366

**Published:** 2026-08-24

**Authors:** Kexin Wang, Kejin Tang, Caixia Liu, Panpan Zhou, Wang He, Ying Xie, Changqing Deng

**Affiliations:** Key Laboratory of Hunan Province for Integrated Traditional Chinese and Western Medicine on Prevention and Treatment of Cardio-Cerebral Diseases, College of Integrated Traditional Chinese and Western Medicine, Hunan University of Chinese Medicine, Changsha 410208, China

**Keywords:** stress-induced vascular senescence, silent information regulator 1, oxidative stress, inflammation

## Abstract

A major focus of contemporary medical research is the primary pathological cardiovascular diseases, whereas stress-induced vascular senescence is a process with degenerative changes in vascular morphology, structure, and function caused by oxidative stress (OS), inflammation, autophagy disorders, and other factors. Silent information regulator of transcription 1 (SIRT1), which may be a key gene linking oxidative stress and aging, has extensive biological functions in anti-oxidation, anti-inflammation, anti-apoptosis, and other aspects. This paper focuses on the protective effects and main mechanisms of SIRT1 on stress-induced vascular senescence and provides clues for preventing and treating diseases related to stress-induced vascular senescence.

## 1 Introduction

The “China Cardiovascular Health and Disease Report 2022” shows that the number of patients with cardiovascular diseases (CVDs), including hypertension, coronary heart disease, stroke, and heart failure, has exceeded 330 million, and age-related CVDs rank first in mortality rate among urban and rural residents. Therefore, stress-induced vascular senescence has attracted widespread attention from clinicians and basic research scholars [1]. Vascular aging includes replicative senescence and stress-induced premature senescence. Replicative senescence refers to irreversible cell-cycle arrest caused by telomere shortening during repeated cell division in the absence of external stress, leading to DNA damage and genomic instability. Stress-induced premature senescence refers to irreversible proliferative arrest caused by specific stressors such as persistent DNA damage, oncogene activation, or oxidative stress [2]. Stress-induced premature aging can be reduced or even reversed by improving the stress environment. Thus, this study mainly examined stress-induced vascular senescence.

Stress-induced vascular senescence affects the occurrence and prognosis of CVDs, and OS is a vital factor that induces CVDs [3], 4]. It can also cause vascular dysfunction, manifested as dilation of the vascular lumen, thickening and stiffening of the vascular wall, and reduced vascular compliance and self-repair ability, ultimately leading to vascular remodeling [5]. Intervention in stress-induced vascular senescence is of great significance for delaying human aging and preventing the onset of age-related chronic diseases. Commonly used noninvasive assessment indicators in clinical practice mainly include intimal thickness testing, arterial stiffness measures (including the pulse wave conduction velocity, ankle-brachial index, cardio-malleolar vascular index, reflex wave enhancement index, large artery elasticity index, and small artery elasticity index), and vascular endothelial function tests, including flow-mediated diastolic function to reflect the occurrence and extent of stress-induced vascular senescence [6].

OS is a pathological state in which the production of reactive oxygen species (ROS) exceeds the capacity of cellular antioxidant systems, resulting in ROS accumulation in cells or tissues. Excess ROS damage DNA, proteins, and lipids and can promote apoptosis. Other contributors to stress-induced vascular senescence include inflammation, renin-angiotensin-aldosterone system (RAAS) activation, autophagy dysregulation, telomere/telomerase alterations, mitochondrial dysfunction, and disrupted nitric oxide (NO) signaling [7], 8]. Studies have found that SIRT1 can delay cellular senescence and ameliorate stress-induced vascular senescence through multiple pathways, including antioxidant effects, anti-inflammatory activity, and reduced apoptosis [9].

This review examines stress-induced premature senescence of the vascular wall, with a primary focus on vascular endothelial cells (VECs); it also incorporates evidence from vascular smooth muscle cells (VSMCs) and whole-vessel models to clarify the multicellular contributions to stress-induced vascular senescence. It discusses the key stressors that drive pathological vascular aging, including oxidative stress, chronic inflammation, autophagy dysregulation, telomere attrition, mitochondrial dysfunction, reduced NO bioavailability, and RAAS hyperactivation. It emphasizes mechanistic *in vitro* and *in vivo* studies of SIRT1 signaling, rather than clinical trial data, to clarify the underlying molecular pathways. Pathways and regulatory factors were selected based on recurrent hub nodes identified through SIRT1 protein-protein interaction network analysis and based on mechanisms repeatedly confirmed in peer-reviewed literature, ensuring a focused, evidence-based structure.

## 2 Predisposing factors for stress-induced vascular senescence

**Figure 1:**
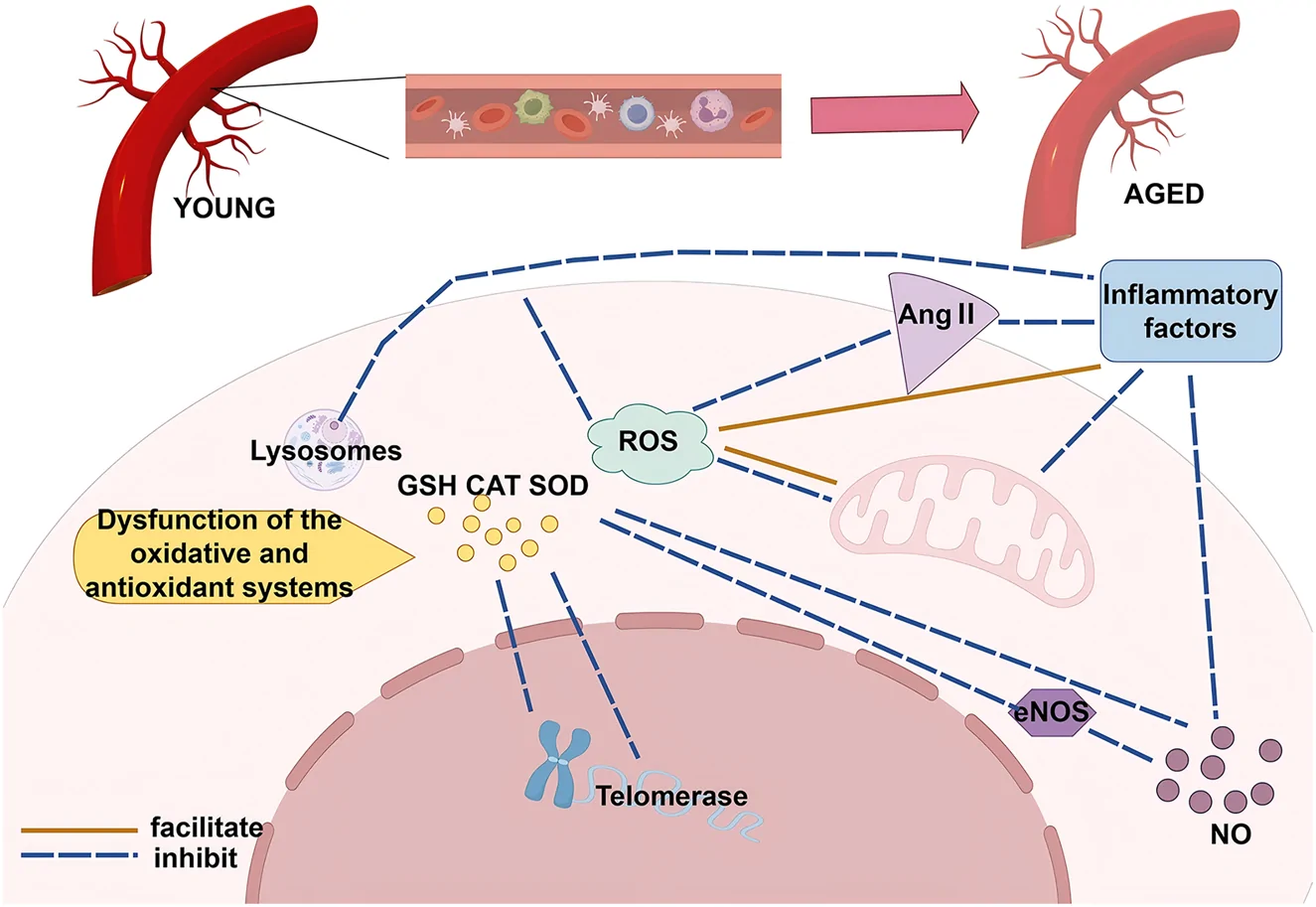
Factors contributing to stress-induced vascular senescence.

Blood vessels are composed of the intima, media, and adventitia, and the main cell types include VECs, VSMCs, and fibroblasts. Damage to any vessel layer or cell type can contribute to stress-induced vascular senescence [10]. VECs form a single layer of flat squamous epithelial cells lining the inner wall of blood vessels, serving as a protective barrier for material exchange between blood and tissues. They regulate biological processes such as oxidative stress, inflammation, vasomotor function, and coagulation by maintaining the integrity of the vascular system, and they are an important link in preserving vascular and internal environmental homeostasis [11]. VEC dysfunction, which has been extensively studied, is a pathological basis closely related to stress-induced vascular senescence that can cause blood flow disorders [12], impair vascular repair and regeneration, reduce the permeability of vascular endothelial cells, and damage the integrity of the cardiovascular system by affecting oxidative stress, inflammatory responses, autophagy, and other functions [13]. The main inducible factors of stress-induced vascular senescence are summarized in Figure 1.

### 2.1 OS

OS is a pathological condition in which the overproduction and impaired elimination of ROS can damage cellular DNA and proteins, destroy the cell membrane and membranous organelles, and induce cellular aging or even death, forming the pathological basis for atherosclerosis, heart failure, arterial hypertension, and other CVDs [4], [14], [15], [16]. As an important second messenger in the body, ROS participate in the transduction of intracellular signals in various biological processes. Its excessive accumulation, triggered by exogenous factors such as radiation, certain drugs, high-sugar foods, and tobacco or endogenous factors such as mitochondrial dysfunction and autophagy disorders can trigger OS, directly damage cellular DNA and proteins, and induce VEC dysfunction [14], [15], [16]. Moreover, if the body exhibits abnormalities in scavenging enzyme systems such as superoxide dismutase (SOD), catalase (CAT), and glutathione (GSH), ROS cannot be cleared normally, resulting in their abnormal accumulation [17].

Accumulated ROS can modify the molecular structure of cellular DNA, proteins, and lipids, disrupt the phospholipid bilayer, and cause damage to cell membranes, membranous organelles, and genomes, resulting in tissue and organ damage [18]. Abnormal ROS levels can also alter the activation state of endothelial nitric oxide synthase (eNOS), reduce the production and bioavailability of NO, impair the body’s ability to regulate vasomotion, inflammation, and thrombosis, and promote the formation of the atherosclerotic vascular phenotype during aging, which is an important mechanism driving cellular aging [19]. OS can also cause telomere shortening, resulting in genomic instability and leading to cell apoptosis and necrosis [20]. OS activates redox-sensitive cell signaling pathways, induces the expression of numerous pro-inflammatory factors and mediators, inhibits the expression of anti-inflammatory factors, and aggravates stress-induced vascular senescence secondary to inflammatory injury [21]. VEC dysfunction caused by OS affects coronary artery dilation and induces neurovascular uncoupling, which may contribute to the development of heart and brain diseases [22]. The generation of ROS is the basis of persistent oxidative stress and is involved in the pathogenesis of many CVDs. OS-induced, stress-induced vascular senescence involves multiple pathways and channels. Endothelial cells in the intima, VSMCs in the media, and macrophages in the adventitia can generate superoxide anion radicals through different pathways, thus promoting vascular oxidative stress responses [13], 23]. Most of the above-mentioned conclusions are supported by abundant *in vitro* and animal experimental evidence, while direct clinical data on human vascular aging are still lacking.

### 2.2 Inflammation

Inflammation is the physiological response of organisms to harmful stimuli. By triggering the production of numerous inflammatory factors, such as chemokines, cyclooxygenase-2, cytokines, and pro-inflammatory transcription factors, the invasion of harmful stimuli can be mitigated, but a persistent inflammatory response can lead to cell and tissue damage [24]. When the body is exposed to harmful stimuli, the expression of pro-inflammatory genes associated with vascular endothelial cells and smooth muscle cells is upregulated, and large numbers of white blood cells are mobilized to infiltrate the vascular wall, creating a pro-inflammatory microenvironment that can readily cause apoptosis of vascular wall cells and induce vascular dysfunction [25]. Inflammatory factors produced during inflammation can exhaust the adaptive immune response, leading to immune aging, which may combine with the inflammatory response to create a vicious cycle that ultimately worsens tissue damage. Many factors, such as DNA damage, organelle dysfunction, autophagy defects, and stem cell aging, can promote inflammation, which is closely associated with oxidative stress [26]. Excess ROS regulate the production of tumor necrosis factor-α (TNF-α), interleukin (IL)-1β, IL-6, and other inflammatory molecules, leading to secondary inflammatory responses and inflammatory damage [27].

Beyond local vascular cytokines detected in tissue specimens, the concept of inflammaging emphasizes a systemic, chronic, low-grade inflammatory state as a key driver of stress-induced vascular senescence, with measurable circulating inflammatory biomarkers serving as translational surrogates for assessing the severity of stress-induced vascular senescence and cardiovascular prognosis [28]. Unlike individual inflammatory cytokines such as IL-6 and TNF-α, composite systemic immune-inflammatory indices derived from routine blood cell counts (including the systemic immune-inflammation index (SII), CRP/albumin ratio (CAR), and uric acid/albumin ratio (UAR)) allow standardized quantitative stratification of the overall inflammatory burden. Longitudinal cohort analyses have confirmed that elevated composite inflammatory markers are independently associated with increased arterial stiffness and carotid intima-media thickness, two classic non-invasive indicators of premature stress-induced vascular senescence [29]. Sustained systemic inflammation accelerates secretion of the endothelial senescence-associated secretory phenotype (SASP), intensifies vascular wall remodeling, and creates a feedforward cycle between inflammatory burden and progressive premature stress-induced vascular senescence [4].

Two clinical studies explicitly confirmed the prognostic value of composite inflammatory indices, as suggested by the peer review comments. First, elevated SII serves as an independent predictive marker for contrast-induced nephropathy in patients with non-ST-segment elevation myocardial infarction; microvascular senescence and endothelial injury caused by systemic inflammatory activation underlie this adverse renal complication following coronary angiography [30]. Second, direct comparisons of multiple inflammatory indices for determining atrial fibrillation recurrence after cryo-ablation show that CAR has better predictive performance than SII and UAR; persistent systemic inflammation promotes chronic atrial stress-induced vascular senescence and structural remodeling, increasing the risk of arrhythmia recurrence in patients with high composite inflammatory index tertiles [31]. Taken together, these clinical observations connect preclinical mechanistic research on inflammation-driven stress-induced vascular senescence with clinical risk stratification, offering measurable laboratory indicators that help translate basic stress-induced vascular senescence mechanisms into practical tools for cardiovascular risk assessment [4], 27].

### 2.3 Autophagy

Autophagy can regenerate metabolic precursors, remove subcellular debris, enhance the quality of organelles and proteins, and maintain cellular homeostasis and integrity under stress. Impairments in autophagy can accelerate the onset and progression of pathological biological processes such as oxidative stress, inflammatory responses, and cell necrosis [32], 33]. Autophagic capacity can decline with age, and a reduced rate of autophagic clearance of poor-quality proteins leads to the continuous accumulation of toxic aggregates in cells, contributing to cellular necrosis and triggering tissue injury [34], 35]. The accumulation of ROS, cytokines, and growth factors in vascular smooth muscle cells can activate autophagy and mitigate damage to these cells [36]. Mitophagy can protect VECs under stress (e.g., hypoxia) by removing damaged mitochondria and reducing the intracellular accumulation of harmful substances such as ROS, thus alleviating VEC injury [37]. However, excessive autophagy can destroy essential organelles and proteins, leading to autophagy-associated cell death and promoting VEC dysfunction [38]. Studies have shown that administering an autophagy enhancer to elderly mice can reduce oxidative stress and inflammation and may even reverse age-related vascular dysfunction [39].

### 2.4 Telomeres and telomerase

Telomeres are complex structures composed of DNA and proteins located at the ends of normal chromosomes. They play an important role in reducing DNA degradation, recombination, and fusion, and they shorten as the number of cell divisions increases [40], 41]. When telomeres shorten to a certain extent, chromosome ends become exposed, triggering a cascade signal similar to DNA damage, which induces inflammatory gene expression and a senescence-associated secretory phenotype [41]. The senescence-associated secretory phenotype of paracrine factors has been shown to induce senescence in neighboring cells [42]. Telomere shortening can be accelerated by senescence, oxidative stress, and inflammatory responses. When telomeres become critically short, the resulting genomic instability activates the DNA repair system and induces cellular replication arrest, senescence, and cell death. In senescent cells, the secretion of pro-inflammatory cytokines, adhesion molecules, growth factors, and other inflammatory mediators is significantly increased, which further worsens telomere dysfunction, promotes replicative aging, creates a vicious cycle, and accelerates the progression of senescence and age-related diseases [43].

### 2.5 Mitochondrial function

Mitochondrial function is impaired by a variety of factors, including the accumulation of mitochondrial DNA mutations, imbalances in the respiratory chain complex, and changes in mitochondrial dynamics, which increase ROS production and trigger alterations in mitochondrial membrane permeability, leading to oxidative stress, inflammation, apoptosis, and stress-induced vascular senescence [44]. ROS attacks on mitochondria lead to increased mitochondrial DNA damage, reduced levels of respiratory chain-related enzymes synthesized by mitochondrial DNA, and a decreased ability of the body to repair the damage, resulting in cell dysfunction [45]. The iron-sulfur center of the electron transport chain in eukaryotic cells can be oxidized by mitochondrial ROS, causing functional damage to the ETC complex and further increasing ROS production [46]. Mitochondrial dysfunction may affect IL-6 signaling through a positive feedback loop, thus influencing inflammatory responses and exacerbating stress-induced vascular senescence [47].

### 2.6 NO

NO, as an endothelium-derived relaxing factor, can dilate blood vessels, scavenge oxygen free radicals, inhibit platelet adhesion, reduce adhesion of inflammatory factors, provide antioxidant, anti-inflammatory, antithrombotic, and blood flow-regulating effects, and mitigate stress-induced vascular senescence. NO stimulates telomerase activity, counteracts the adverse effects of telomere shortening, reduces NO donor erosion, and relieves oxidative stress-induced VEC senescence [48]. VEC dysfunction causes ROS accumulation, which lowers intracellular l-arginine levels and impairs the cofactor activity of eNOS, leading to decreased NO production, disruption of the intravascular environment, and worsening of VEC injury [49].

### 2.7 RAAS

The hyperactivity of RAAS induces OS and chronic inflammatory responses, increases the incidence of vascular atherosclerosis, makes vascular cells more vulnerable to damage, and promotes stress-induced vascular senescence [50]. Angiotensin II expression and activation mediate intimal fiborsis, resulting in the deposition of collagen and fibronectin and the degradation of elastin, which cause structural changes in the vasculature and lead to stress-induced vascular senescence [51]. Abnormal activation of the salt corticosteroid receptor by aldosterone can induce changes in the vascular structure and function through various mechanisms, including effects on oxidative stress, inflammatory responses, and vascular tone [52]. Studies have shown that inhibiting RAAS activity can reduce arterial stiffness in olde r adults and aged animals, regulate changes in blood pressure, and improve the ability of blood vessels to resist aging [53].

## 3 SIRT1 and the sirtuin protein family

The Sirtuin family is a group of NAD + -dependent deacetylases in mammals, including SIRT1 through SIRT7, which are involved in various biological processes such as cell proliferation, cellular senescence, apoptosis, cellular metabolism, gene transcription, and DNA repair [54]. The Sirtuin family is a regulatory factor associated with aging, and it plays a protective role in age-related diseases such as CVDs, neurodegenerative disease, diabetes, and metabolic disease [55]. Members of the Sirtuin family located in the mitochondria include SIRT3, SIRT4, and SIRT5 [53]. SIRT3 can regulate various cellular processes, including growth arrest, apoptosis, aging, and metabolism, and its expression in VECs can regulate the metabolic transition between glycolysis and mitochondrial respiration [56]. SIRT4 has been studied less extensively, and it is more highly expressed in adult leukocytes and the embryonic thymus. It can maintain genomic stability, regulate mitochondrial function, and play an important antioxidant and anti-inflammatory role [57]. SIRT5, which has depropionylation and weak deacetylation activities, participates in the regulation of various physiological processes, including the tricarboxylic acid cycle, urea cycle, glycolysis, gluconeogenesis, and redox homeostasis [58].

Current studies have shown that SIRT6 and SIRT7 are expressed in the nucleus [43]. As an important member of the Sirtuin family, SIRT6 possesses both deacetylase activity and ADP-nucleic acid transferase activity and is highly similar to SIRT1 in function [59]. SIRT6 acts on multiple transcription factors during aging, helps repair and maintain telomeric DNA chromatin structure, and plays an important protective role in maintaining genomic stability [60]. SIRT7, the least-studied sirtuin protein to date, exists only in eukaryotes; and it has important functions in promoting ribosomal RNA expression, repairing DNA damage, and regulating chromatin compression [61].

SIRT2 is the only sirtuin protein predominantly expressed in the cytoplasm, although it is also present in small amounts in the nucleus and mitochondria [53]. SIRT2 is closely involved in a variety of biological processes, including apoptosis, autophagy, and immune responses. It regulates the expression of its target genes during processes such as replication, transcription, and translation. By reducing oxidative stress and inflammatory responses associated with metabolic disorders, SIRT2 plays a protective role in CVDs, neurodegenerative diseases, tumors, diabetes, and other diseases related to metabolic dysfunction [62], 63].

**Figure 2:**
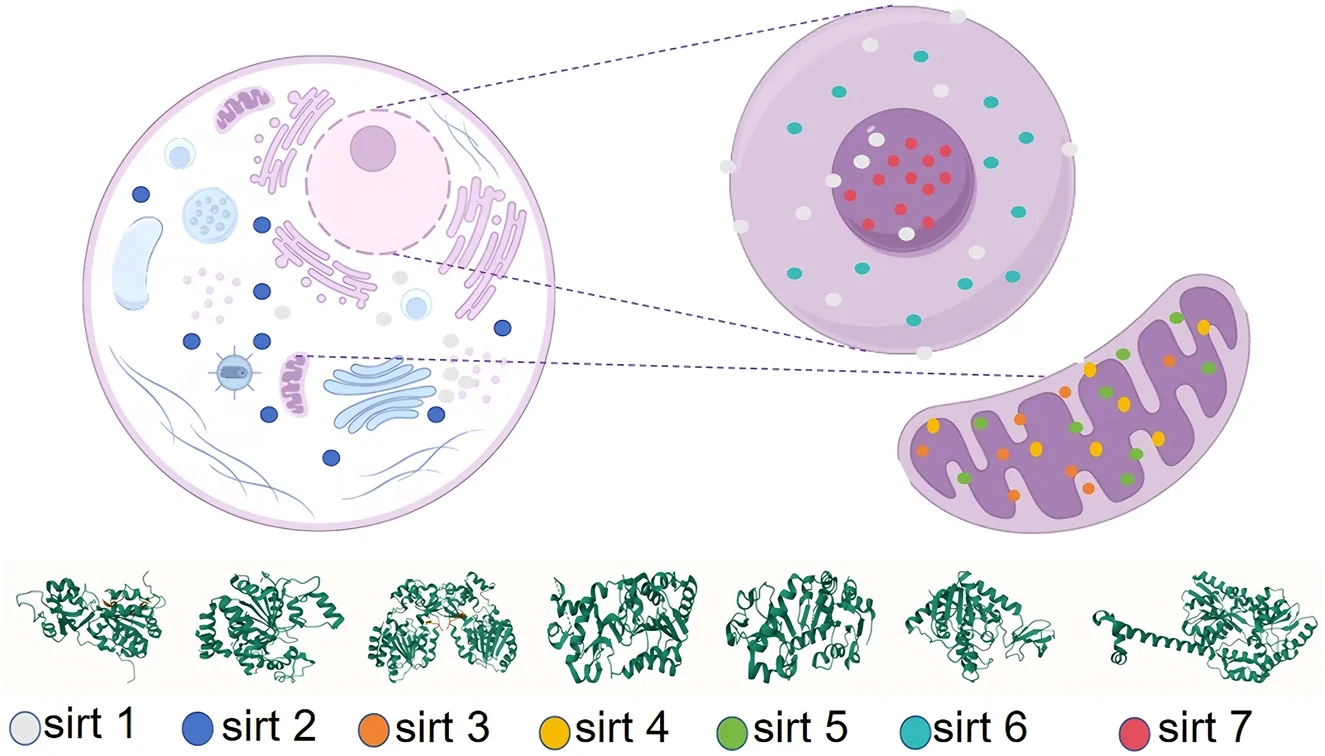
Structures of SIRT1-SIRT7. SIRT1: Primarily located in the nucleus and capable of shuttling between the nucleus and cytoplasm; SIRT2: Mainly distributed in the cytoplasm and translocates to chromosomes during mitosis; SIRT3/4/5: Exclusively present in mitochondria, serving as mitochondria-specific deacetylases; SIRT6/7: Specifically localized in the nucleus, with SIRT6 predominantly binding to chromatin and SIRT7 enriched in the nucleolus.

The SIRT1 gene (Figure 2) is located on human chromosome 10 and spans a total length of approximately 33 kb, containing nine exons, eight introns, and untranslated regions [64]. The conserved catalytic structure of SIRT1 consists of both C-terminal and N-terminal domains. The C-terminal domain complements the β-fold of the NAD + binding domain, while the N-terminal domain is essential for SIRT1 activity [65]. As a longevity-associated protein, SIRT1 is primarily located in the nucleus but is also expressed to a lesser extent in the cytoplasm, where it exhibits NAD + -dependent deacetylase activity. It plays a crucial role in the development of diseases such as cardiovascular, neurodegenerative, and metabolic disorders, linking the aging process to vascular health and metabolic damage [66], 67]. The diversity of SIRT1’s functions depends on the variety of its C-terminal domains, which are involved in regulating biological processes such as oxidative stress, inflammatory response, energy homeostasis, mitochondrial dysfunction, apoptosis, and autophagy [68]. SIRT1 is highly sensitive to the cellular redox state and supports cardioprotection and the maintenance of vascular function by deacetylating a variety of effector molecules and attenuating the effects of ROS [69]. Research on how key members of the sirtuin family regulate CVDs provides effective new approaches for the treatment and prevention of these diseases and is currently one of the main areas of study.

## 4 Network analysis

The STRING database compiles publicly available omics data through the latest updates, covering published genomic, transcriptomic, proteomic, and literature-mined data on human protein interactions. UniProt is an internationally recognized database for protein sequences and functional annotation, containing extensive information on the primary sequences of proteins from numerous species, biological functions, modification sites, interaction relationships, and disease-related data. This study uses the UniProt and STRING databases, integrating data results through April 1, 2026 and encompassing long-term accumulated research on molecular regulation. Using SIRT1, oxidative stress, and inflammatory response as the core search keywords, functional proteins related to oxidative stress and inflammation were retrieved from UniProt, with priority given to proteins directly associated with SIRT1 and stress-induced vascular senescence phenotypes. These were combined with SIRT1 to construct an interaction network, and key targets were selected based on betweenness centrality, retaining high-confidence molecules that were directly bound or indirectly regulated while removing redundant targets with low relevance. Cytoscape was used to complete network visualization and topology analysis.

This study used the STRING V12.0 database, restricting the species to *Homo sapiens* and setting the interaction confidence score threshold to the database’s default high-confidence threshold of 0.7. Evidence channels included experimental validation, database annotation, gene co-expression, and literature text mining. Seven rounds of target expansion were conducted based on node-by-node association rules to comprehensively identify downstream and upstream cascade regulatory targets of SIRT1, fully covering oxidative stress- and inflammation-related signaling pathways while avoiding the omission of key molecules. The maximum number of interacting proteins per round was limited to 20, and SIRT1’s direct and indirect regulatory molecules were mined layer by layer to clarify the logic of multi-level molecular regulatory networks. The resulting protein interaction network was imported into Cytoscape software, and topological analysis was conducted using the CytoNCA plugin. In the software’s Analyze Network function, betweenness centrality was chosen as the core evaluation metric, and the Pearson’s correlation coefficient of protein interactions was used to evaluate the closeness of molecular associations. Nodes were ranked by descending correlation coefficient, with node color intensity and size representing the strength of interactions.

Based on the UniProt 2026-01 database, target proteins were identified using standard GO functional terms for oxidative stress and inflammatory response together with official keywords. The selection criteria were restricted to human-derived, reviewed (Swiss-Prot), high-quality annotated proteins, excluding low-confidence unannotated sequences and unrelated cross-species proteins. The screened proteins related to oxidative stress and inflammation were co-imported with SIRT1 into STRING to build a specific regulatory network. Node association weights and centrality parameters were recalculated in Cytoscape to finalize the hierarchical visualization analysis.

Gene Ontology (GO) enrichment analysis provides a straightforward annotation of gene products. The selected SIRT1-related genes were entered into the DAVID database, with “*H. sapiens*” selected as the organism, and GO analysis was carried out according to the biological process (BP), cellular component (CC), and molecular function (MF). Kyoto Encyclopedia of Genes and Genomes (KEGG) enrichment analysis emphasizes the interaction networks of molecules within the organism. Through this analysis, the signaling pathways of target molecules can be identified. Relevant information for KEGG enrichment analysis was obtained from DAVID. Based on the p-values, the top 10 data points with the smallest values were selected. After organizing the data, microinformatics was used for visualization. As shown, A denotes a BP, B denotes a CC, C denotes an MF, and D denotes KEGG pathway analysis.

### 4.1 Analysis of the protein interaction network

**Figure 3:**
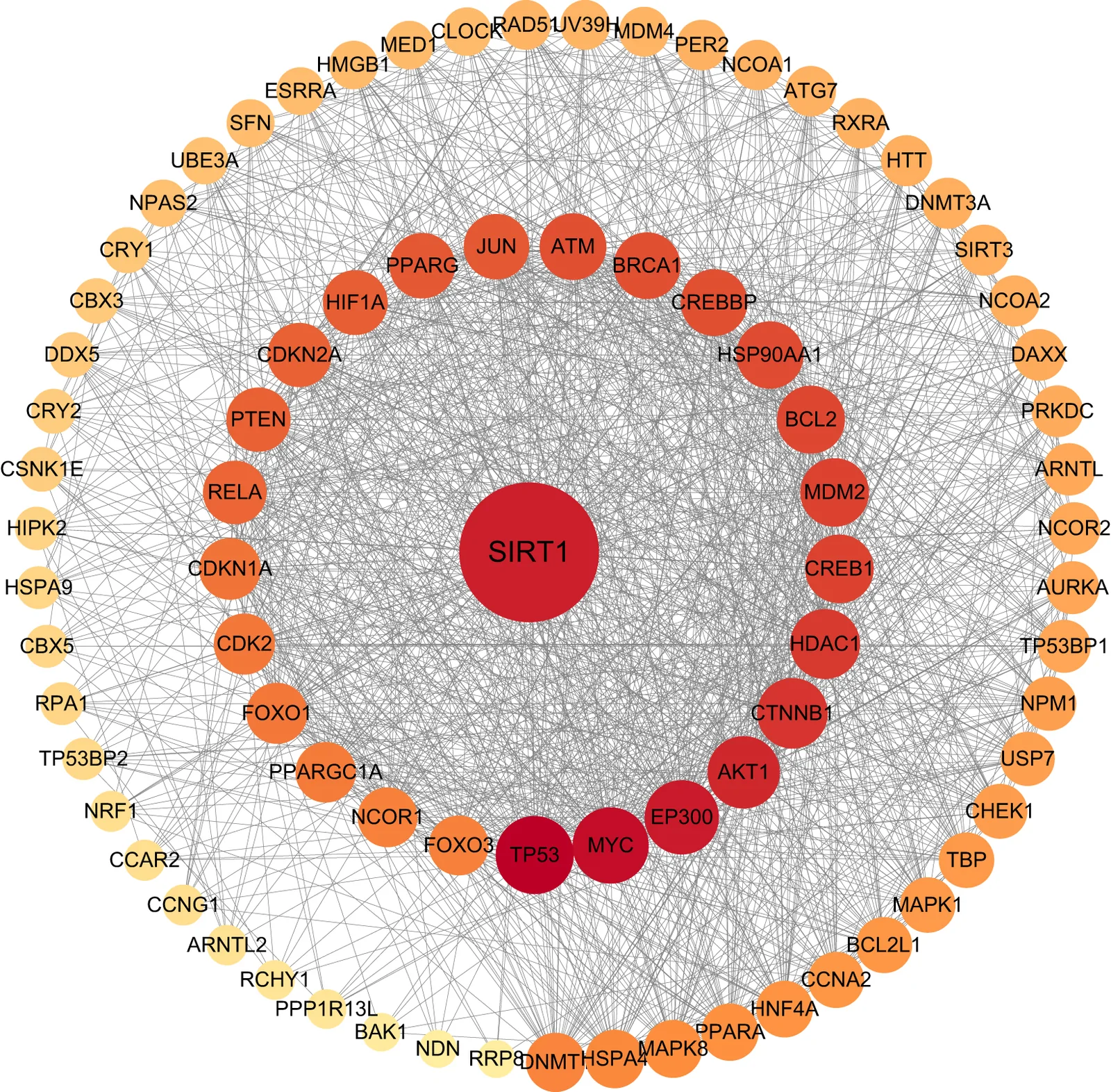
Global protein-protein interaction network of SIRT1.

**Figure 4:**
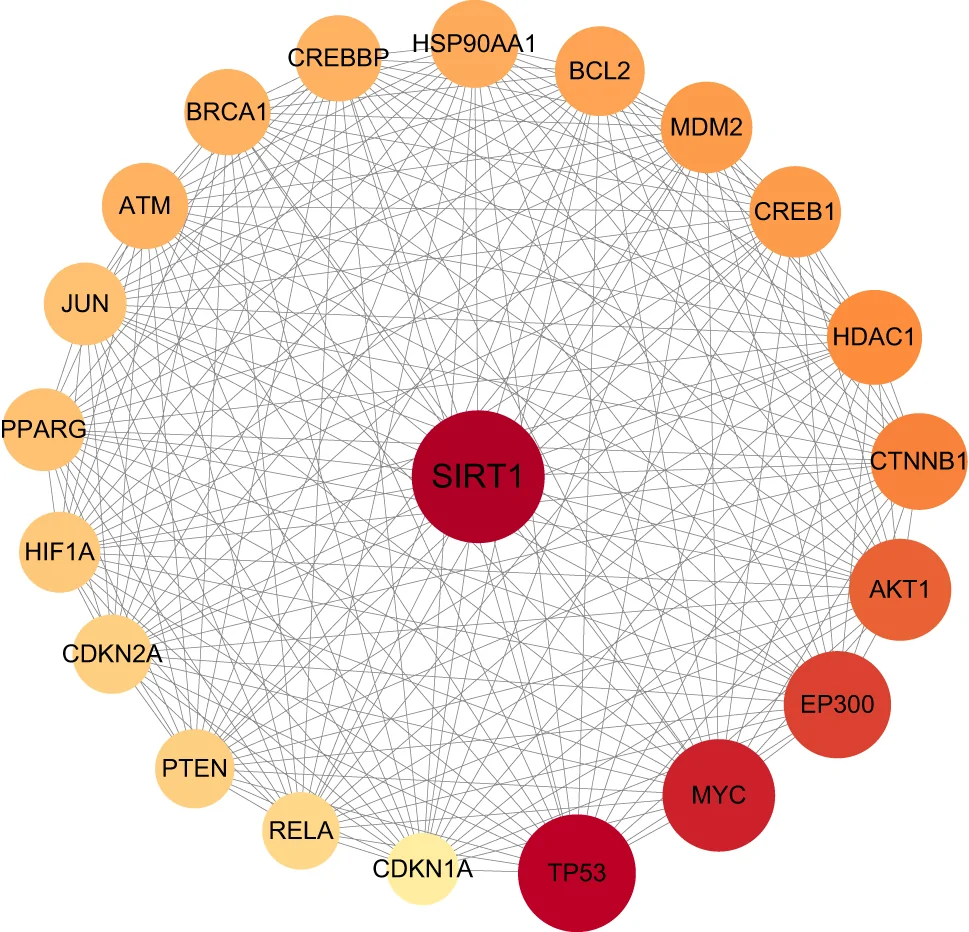
Screening of core targets in the protein interaction mapping of SIRT1.

By searching the STRING database platform, we constructed a protein-protein interaction network for “SIRT1” to identify protein targets that interact with it directly or indirectly. The STRING database was queried by entering “SIRT1” and setting the species to “*H. sapiens*” while keeping the default parameters unchanged. After seven rounds of expansion, 80 interacting proteins were identified. The protein interaction network was then imported into Cytoscape for calculation and analysis. The protein interactions were imported into Cytoscape, and the molecules were arranged counterclockwise according to their correlation coefficients. For a redder color of the molecule, the area is larger, and the correlation with other protein molecules is stronger (Figure 3). Among them, 62 molecules (77.5 %) were directly related to SIRT1, and 18 molecules (22.5 %) were indirectly related. There were 35 molecules related to oxidative stress (44 %), primarily including “EP300,” “CREB1,” “HDAC1,” “NCOR1,” “FOXO1,” “JUN,” “NRF1,” “BCL2,” “CDKN1A,” “TP53,” “SUV39H1,” “BRCA1,” “MDM2,” “NCOR2,” “PPARG,” “PPARA,” “PPARGC1A,” “HMGB1,” “ESRRA,” and “MAPK8”. There were 27 (33.6 %) molecules related to inflammation, mainly including “EP300,” “PPARG,” “CTNNB1,” “CREB1,” “NDN,” “AKT1,” “HDAC1,” “HMGB1,” “RELA,” “FOXO1,” “MAPK8,” “MAPK1,” “JUN,” “HSP90AA1,” “HSPA9,” “BCL2,” “CREBBP,” “NCOA1,” “CDKN1A, and “FOXO3.” The network interaction graph was further analyzed using the CytoNCA tool in Cytoscape, with proteins color-coded and arranged counterclockwise according to betweenness centrality. The top 20 proteins ranked by betweenness centrality were selected for plotting, revealing that SIRT1 had the highest betweenness centrality. Among these top 20 proteins, 16 were associated with oxidative stress, and 14 were associated with inflammation (Figure 4).

### 4.2 Cytoscape analysis

**Figure 5:**
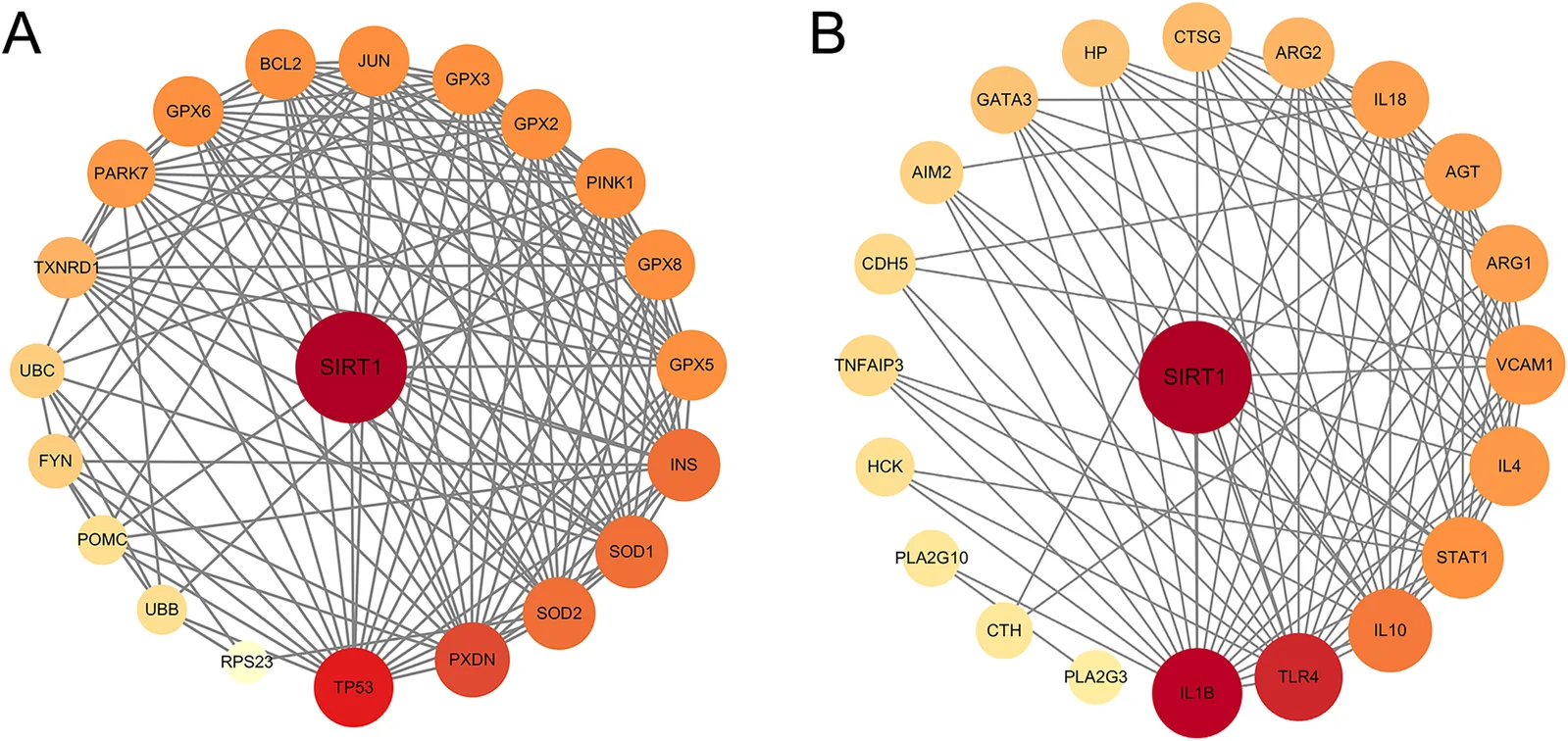
Protein-protein interaction map of SIRT1 (A involved in oxidative stress; B involved in inflammation).

Searching the UniProt platform using the term “Oxidative stress” yielded proteins associated with oxidative stress, and these data were entered into STRING to construct a protein-protein interaction network. The network was then imported into Cytoscape, where the top 20 oxidative stress-related proteins ranked by betweenness centrality were identified. These proteins, together with SIRT1, were simultaneously entered into STRING to obtain a protein interaction network map, which was imported into Cytoscape to calculate correlation coefficients and rank them counterclockwise by correlation (Figure 5A). Protein molecules directly related to SIRT1 included “BCL2,” “INS,” “PINK1,” “TP53,” “SOD2,” “SOD1,” “GPX6,” “GPX2,” “GPX3,” “GPX5,” “GPX8,” “PXDN,” and “JUN,” accounting for 65 %. Molecules indirectly related to SIRT1 included “TXNRD1,” “UBB,” “UBC,” “RPS23,” “PARK7,” “FYN,” and “POMC,” accounting for 35 %. We searched the UniProt platform and entered “inflammation” to identify inflammation-related proteins and then entered the related proteins into STRING to obtain the protein interaction network. This network was imported into Cytoscape, and the top 20 proteins associated with inflammation were identified and ranked by betweenness centrality. These proteins, together with SIRT1, were entered into STRING to generate an interaction network, which was imported into Cytoscape for correlation coefficient calculations and arranged counterclockwise according to relatedness (Figure 5B). SIRT1 was directly associated with molecules such as “IL4,” “IL18,” “AGT,” “VCAM1,” “IL1B,” “IL10,” “TLR4,” and “STAT1,” accounting for 40 %, and indirectly associated with “ARG2,” “PLA2G3,” “CDH5,” “GATA3,” “CTSG,” “ARG1,” “CTH,” “TNFAIP3,” “HCK,” “PLA2G10,” “HP,” and “AIM2,” among others, accounting for 60 %.

### 4.3 Gene ontology and KEGG pathway enrichment analyses

**Figure 6:**
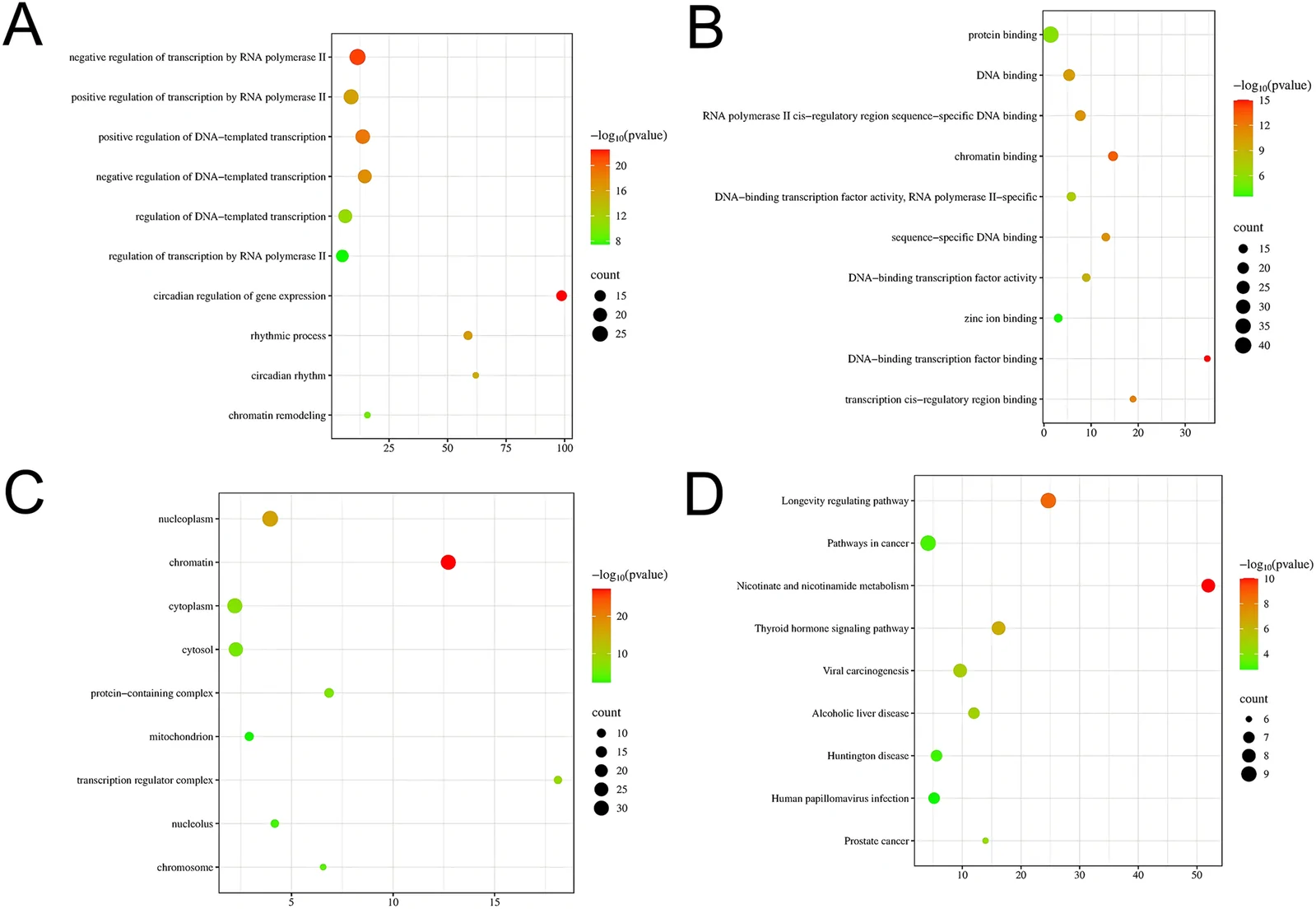
SIRT1 enrichment bubble chart (A shows BP involvement; B shows MF involvement; C shows CC involvement; and D shows KEGG involvement).

The GO analysis results showed that the numbers of target genes interacting with SIRT1 classified into BP, MF, and CC were 191, 90, and 33, respectively. These target genes were mainly distributed in the plasma membrane, cytoplasm, and nucleus; they were primarily involved in protein binding, DNA binding, and RNA polymerase II regulation; and they participated in BPs such as signal transduction, positive regulation of transcription, and the inflammatory response. KEGG pathway enrichment analysis revealed that SIRT1 is closely associated with stress-induced aging signaling pathways (Figure 6).

## 5 SIRT1 regulates signaling pathways associated with stress-induced vascular senescence

**Figure 7:**
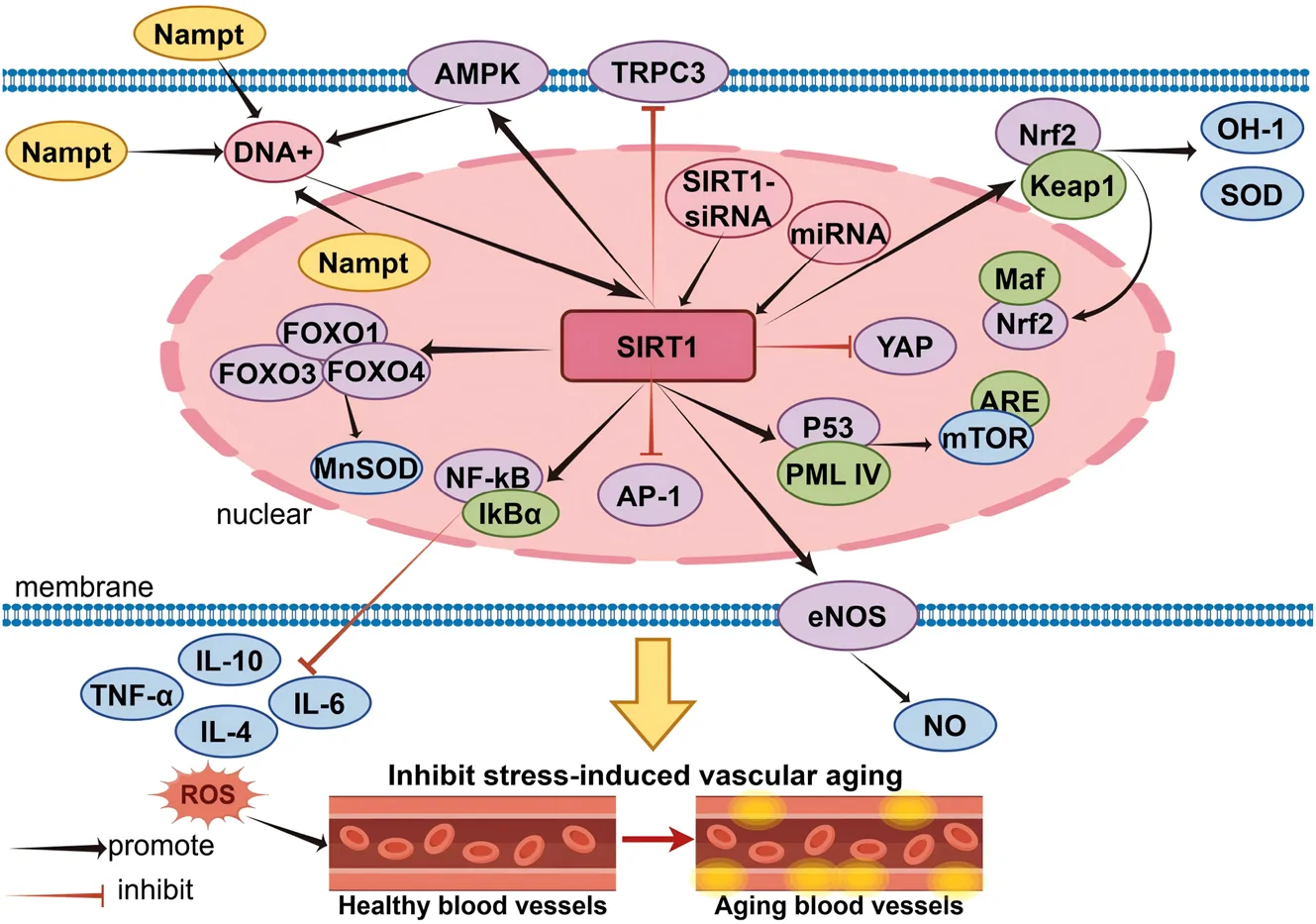
SIRT1 pathway diagram.

SIRT1 can mitigate stress-induced vascular senescence by activating or inhibiting multiple effector molecules in the body, regulating the activity of downstream molecules, and exerting antioxidant and anti-inflammatory effects. Figure 7 illustrates the network diagram of SIRT1-related signaling pathways, and the main effector molecules include Nrf2, FoxOs, NF-κB, P53, eNOS, HIF-1α, AMPK, P66Shc, and AP-1 (Table 1).

**Table 1:** SIRT1 pathways regulating irritant stress-induced vascular senescence.

Study ID	Main related signaling pathways	Effector molecules	Mechanism of action	Modeling approach	Ref.
1	miR-126/SIRT1/Nrf2/HO-1, SOD, VCAM-1, MCP-1, TNF-α	SIRT1, Nrf2, HO-1, SOD↑ VCAM-1, MCP-1, TNF-α, ROS↓	(−) oxidative stress (−) inflammation	Ligation of the left anterior descending coronary artery in SPF male Wistar rats	[73]
2	SIRT1/FoxOs/MnSOD, SOD2, CAT	SIRT1, FoxO1, Rab7, FoxO4, FoxO3, MnSOD, SOD2, CAT↑ ROS↓	(−) autophagy (−) oxidative stress (−) endothelial damage	SPF male SD rats undergo permane-nt ligation of both common carotid arteries	[76]
3	miR-9/SIRT1/NF-κB/TNF-α, IL-6, IL-4, IL-1β, IL-10	SIRT1↑ NF-κB, TNF-α, IL-6, IL-4, IL-1β, IL-10, ROS↓	(−) oxidative stress (−) inflammation	miR-NC and/or miR-9 inhibitor were transfected into human myocardial H9c2 cells	[77]
4	Nampt, FGF1, heat shock protein 25/SIRT1/P53/PML IV, Ras, mTOR	Nampt, FGF1, heat shock protein 25SIRT1, mTOR↑ p53, PMLIVRas↓	(−) Apoptosis (−) oxidative stress	Six- to seven-week-old SPF male SD rats were intraperitoneally injected with a D-galactose solution (500 mg/kg).	[78], [79], [80], [81], [82]
5	microRNA-217, miR-34a/SIRT1/eNOS/NO	miR-34a, SIRT1, eNOS, NO↑ microRNA-217, ROS↓	(−) apoptosis (−) oxidative stress	SD rat ligation/perfusion of the left anterior descending coronary artery	[82], [83], [84]
6	SIRT1/HIF-1α/VEGFA	SIRT1↑ HIF-1α, VEGFA, ROS↓	(−) oxidative stress	Sirt1endo+/− mice were mated with Sirt1Flox/Flox mice	[85]
7	C1q/tumor necrosis factor-related protein 3SIRT1/AMPK/NF-κB, AP-1	AMPK, C1q/tumor necrosis factor-related protein 3SIRT1↑ ROS, NF-κB, AP-1↓	(−) oxidative stress (−) inflammation	SPF-grade db/db mice	[86]
8	SIRT1/P66Shc/H3, FOXO3a, MnSOD	SIRT1, FOXO3a, MnSOD↑ H3, P66Shc↓	(−) endothelial damage	Six-week-old SPF male C57BL/6 mice were fed a high-fat diet	[87]
9	SIRT1/AP-1/COX-2, PGE2	SIRT1↑ COX-2, PGE2↓	(−) inflammation	Male C57BL/6 mice had 60 % caloric restriction.	[88]
10	SIRT1/TRPC3/TGF-β	SIRT1↑ TRPC3, TGF-β↓	(−) fibrosis	Male C57B6 mice fed a high-Hcy diet	[89]
11	SIRT1/YAP	SIRT1↑ YAP↓	(−) growth	Female ApoE^−/−^ mice aged 4–6 weeks on a Western chow diet	[90]

### 5.1 miR-126/SIRT1/Nrf2/HO-1, SOD, VCAM-1, MCP-1, and TNF-α

The leucine transcription factor nuclear factor erythroid 2-related factor 2 (Nrf2), a major regulator of the body’s antioxidant and anti-inflammatory responses, can correct intracellular redox imbalance and reduce pro-inflammatory factors and mediators through downstream target genes and enzymes [70]. In exploring the mechanism by which YiXinFang improves myocardial injury after ischemia/reperfusion in rats, Dong Li et al. found that YiXinFang significantly increased the levels of SIRT1 and Nrf2 in rat tissues and affected the expression of Nrf2 downstream molecules, indirectly suggesting that SIRT1 may help regulate the expression of Nrf2 and its downstream molecules [71]. A high-sugar diet promotes miR-221 overexpression, which downregulates the expression of heme oxygenase 1 (HO-1) and SOD by inhibiting the SIRT1/Nrf2 pathway, leading to impairment of the body’s antioxidant system [72]. Under oxidative stress, Nrf2 separates from Kelch-like ECH-associated protein 1 (Keap1) and becomes activated. SIRT1 can further enhance Nrf2 activity, promoting its binding to Maf/ARE elements and increasing the transcription of antioxidant target genes [73]. Upregulation of Nrf2 expression or activity in endothelial cells reduces the expression of pro-inflammatory cytokines. The expression levels of vascular cell adhesion protein 1 (VCAM-1), monocyte chemoattractant protein-1 (MCP-1) and TNF-α were also reduced, thus alleviating inflammatory responses [74]. Dysregulation of the SIRT1/Nrf2 signaling pathway has been implicated in the pathogenesis of numerous neurodegenerative diseases, and several studies have shown that overexpression of miR-126 effectively mitigates oxygen-glucose deprivation/reoxygenation-induced oxidative stress and inflammation by activating the SIRT1/Nrf2 signaling pathway [75]. Evidence supporting this pathway is derived primarily from *in vitro* endothelial cell experiments and *in vivo* rat models, with oxidative stress and inflammation serving as the main endpoints. These findings provide only indirect evidence for stress-induced vascular senescence and lack direct validation in human stress-induced vascular senescence models.

### 5.2 SIRT1/FoxOs/Rab7, MnSOD, SOD2, and CAT

Forkhead transcription factor (FoxO) is a transcription factor produced by SIRT1 deacetylation. As one of the most widely studied Forkhead transcription factors, FoxO belongs to a subgroup of the Forkhead family and plays an important role in resisting oxidative stress and preventing endothelial cell damage [75]. The binding of SIRT1 to FoxO1 enhances the ability of FoxO1 to bind DNA, induces the accumulation of FoxO4 in the nucleus, reduces damage to antioxidant-related genes, and enhances the activity of the antioxidant system by inhibiting FoxO1 phosphorylation. It also inhibits the accumulation of ROS in tissues and alleviates oxidative stress [91], [92], [93]. When verifying whether Pueraria baicalensis Lian Tang has antioxidant capacity, Zhang Yuanyuan et al. found that it could upregulate the expression of AMPK, SIRT1 mRNA, and related proteins in liver tissues, while SIRT1 could help ameliorate oxidative stress in the livers of db/db mice by downregulating FoxO1 gene expression and reducing ROS generation [94]. Deacetylation of Forkhead box O3 (FOXO3) by SIRT1 enhances FOXO3-induced cell cycle arrest and inhibits FOXO3-mediated apoptosis [95]. This pathway is supported mainly by *in vivo* rat studies and *in vitro* cell assays, with a focus on oxidative stress and endothelial damage. Evidence for stress-induced vascular senescence remains indirect.

### 5.3 miR-9/SIRT1/NF-kappa B/TNF-alpha, IL-6, IL-4, IL-1β, and IL-10

Nuclear factor kappa-B (NF-κB) plays an important role in regulating oxidative stress, the inflammatory response, the cell cycle, and apoptosis [96]. While studying the role of miR-9 in TNF-α-induced cardiomyocyte injury, Zhang Zhaohua et al. identified SIRT1 as the target gene of miR-9 through bioinformatics analysis and transfected cardiomyocytes with a miR-9 inhibitor to reduce miR-9 expression in these cells. The results showed that knocking down miR-9 expression not only significantly increased SIRT1 protein expression in cardiomyocytes but also decreased NF-κB protein phosphorylation and significantly reduced TNF-α-induced oxidative stress injury in cardiomyocytes, suggesting that miR-9 may inhibit activation of the NF-κB signaling pathway by suppressing SIRT1 protein expression, thus reducing cellular activity and inducing stress-induced vascular senescence [86].

By deacetylating NF-κB, SIRT1 enables NF-κB to bind to its nuclear inhibitor (inhibitor kappa B alpha, IκBα), significantly reducing NF-κB activity, downregulating the expression of TNF-α, IL-6, IL-4, IL-1B, IL-10, and other pro-inflammatory cytokines, decreasing the production of ROS in the body, and promoting ROS elimination by the antioxidant system. This helps maintain the body’s oxidative balance, reduces vascular damage caused by oxidative stress, and serves to alleviate stress-induced stress-induced vascular senescence [76]. Current evidence is mostly derived from *in vitro* cardiomyocyte models and extrapolated to the vascular endothelium. There is insufficient direct evidence for stress-induced vascular senescence.

### 5.4 Namp, FGF1, heat shock protein 25/SIRT1/P53/PMML IV, Ras, and mTOR

p53 is a non-histone substrate of Sirt1 and a core molecule that regulates apoptosis and prevents irritant stress-induced vascular senescence through anti-oxidation and the regulation of apoptosis [97]. Nicotinamide phosphoribosyltransferase (Nampt) can increase intracellular NAD + content, enhance SIRT1 activity, and reduce the production of acetyl-p53 (Lys382) [77]. SIRT1 acts on the histone target H3K9 in the promoter region of the P53 gene, reducing H3K9 acetylation and increasing its trimethylation, thus inhibiting p53 transcription [98]. Abnormal expression of promyelocytic leukemia protein IV (PML IV) or rat sarcoma (Ras) in the organism can cause massive recruitment of SIRT1 to the nucleus, directly affecting PML IV and p53 and interfering with PML-induced cellular senescence by inhibiting p53 acetylation [99]. p53 also activates upstream regulators of mammalian target of rapamycin (mTOR) to increase cellular autophagy and mitigate irritant stress-induced vascular senescence [78]. Supporting data originate mainly from *in vivo* animal models, with apoptosis and DNA damage as key outcomes. These are related to, but are not direct markers of, stress-induced vascular senescence.

### 5.5 miR-34a, microRNA-217, and SIRT1/eNOS/NO

Previous studies have identified a specific binding site between miR-34a and Sirt1. The results showed that, compared with the control group, Sirt1 expression in the myocardial tissue of mice in the Gm44981 overexpression group was upregulated, while miRNA-34a expression was reduced, suggesting that Gm44981 may inhibit cardiac aging by acting on the miRNA-34a/Sirt1 pathway [79].

eNOS is one of the most important enzymes regulating endothelial function, and it is mainly expressed in endothelial cells. It can inhibit ROS production and upregulate NO expression, thus regulating endothelial function, reducing oxidative stress, and improving stress-induced vascular senescence [80]. microRNA-217 can inhibit SIRT1 expression and eNOS deacetylation and promote endothelial cell senescence [81]. On the one hand, SIRT1 can influence eNOS protein expression by regulating the eNOS gene promoter. On the other hand, SIRT1 can enhance NO production by regulating eNOS activity through deacetylation, thus alleviating stress-induced vascular senescence [82]. This pathway is supported by *in vitro* endothelial studies and *in vivo* rat models, with partial confirmation from human vascular function assays. It represents the most consistent and clinically relevant evidence among all the pathways.

### 5.6 SIRT1/HIF-1α/VEGFA

Hypoxia-inducible factor-1α (HIF-1α) is the primary regulator of oxygen homeostasis, and it plays an important role in angiogenesis, energy metabolism, cell survival, apoptosis, and the maintenance of cellular stability under hypoxic conditions [83]. SIRT1 can reduce the accumulation of HIF-1α in the body by inhibiting HIF-1α gene transcription and deacetylating HIF-1α, thus maintaining mitochondrial and oxidative homeostasis and exerting antioxidant effects to prevent stress-induced vascular senescence [84], 100]. SIRT1 may reduce erythropoietin-mediated pathological blood vessel regeneration by inhibiting the HIF-1α signaling pathway and downregulating the expression of vascular endothelial growth factor A (VEGFA) and vascular endothelial growth factor R2 [101], 102]. The evidence is based on genetically modified mouse models and focuses on hypoxia and angiogenesis. Its connection to stress-induced vascular senescence is indirect.

### 5.7 C1q/tumor necrosis factor-related protein 3/SIRT1/AMPK/NF-κB, AP-1

Adenosine 5′-monophosphate (AMP)-activated protein kinase (AMPK) is a key kinase that regulates cellular energy and mitigates stress-induced vascular senescence through anti-inflammatory and antioxidant effects [103]. SIRT1 also deacetylates the serine/threonine kinase hepatic kinase B1, thus activating AMPK, and the effects of SIRT1 and AMPK can be enhanced by a reciprocal positive regulatory circuit [104]. Through SIRT1, AMPK can downregulate the acetylation levels of NF-KB, AP-1, histones, and other target factors, reduce the expression of inflammation-related gene pathways, and alleviate inflammatory damage [85]. Similarly, activation of SIRT1 through complement C1q/tumor necrosis factor-related protein 3 can exert an anti-inflammatory effect by reducing tumor necrosis sub-α levels and NF-κB nuclear translocation. Studies have found that red sandalwood can alleviate mitochondrial damage and oxidative stress by upregulating the expression and deacetylation of PGC-1α through the AMPK and SIRT1 cascade reaction [105]. These findings are mainly derived from diabetic mouse models, with anti-inflammatory and antioxidant effects as the primary endpoints. Extrapolation to general stress-induced vascular senescence requires caution.

### 5.8 SIRT1/P66Shc/H3, FOXO3a, and MnSOD

SHC articulin p66 (generic shell script compiler, P66Shc) is a member of the Shc articulin family of proteins, and inhibition of P66Shc expression has a protective effect on vascular endothelial cells; and it delays stress-induced vascular senescence [106]. By binding to the P66Shc promoter, SIRT1 reduces the acetylation of histone H3 and inhibits the transcription of P66Shc [107]. Under oxidative stress, p66Shc can be transferred to the nucleus and transported to mitochondria and related membranes; and it can bind to mitochondrial cytochrome c, resulting in the oxidation of cytochrome c and further promoting ROS production [108]. However, phosphorylation and activation of p66Shc can activate AKT, thus inactivating the FOXO3a transcription factor, reducing MnSOD levels, and decreasing the body’s ability to clear ROS [109]. Supporting evidence is based on high-fat diet mouse models that measure ROS and endothelial injury. However, these are surrogate phenotypes rather than direct markers of stress-induced vascular senescence.

### 5.9 SIRT1/AP-1/COX-2, PGE2

Activator protein-1 (AP-1) is an important transcriptional activator of leucine zipper DNA-binding proteins; and it can mitigate the negative effects caused by cytokines, chemokines, oncogenic proteins, and other molecules by regulating the expression of downstream genes [110]. High expression of SIRT1 can exert anti-inflammatory effects by regulating the AP-1 gene, downregulating the expression of cyclooxygenase-2 (COX-2) protein in macrophages, and reducing the production of prostaglandin E2 (PGE2) [87]. Evidence is limited to calorie-restricted mouse models and is centered on anti-inflammatory effects. The direct relevance to stress-induced vascular senescence is weak.

### 5.10 SIRT1/TRPC3/TGF-β

Short transient receptor potential channel 3 (TRPC3) is a key factor in regulating myocyte fibrosis; and it may contribute to mechanical stress-induced LV diastolic dysfunction by indirectly regulating TRPC3-mediated expression of the pro-fibrotic protein transforming growth factor-β (TGF-β), and TRPC3 can also negatively regulate SIRT1 expression by directly interacting with SIRT1 [111]. Blocking TRPC3 expression or reducing it through knockdown can decrease Ang II-induced migration, proliferation, and the expression of fibrosis biomarkers in atrial fibroblasts. Meanwhile, the Ca^2+^-permeable TRPC3 channel mediates the atrial fibrosis signaling pathway through Ca^2+^, suggesting that this pathway may be a potential therapeutic target in the pathogenesis of AF during aging and hypertension [112]. This pathway is supported by hyperhomocysteinemic mouse models focused on vascular fibrosis. Evidence for senescence is indirect.

### 5.11 SIRT1/YAP

Yes-associated protein (YAP) is a potential therapeutic target for atherosclerosis. It acts as a sensor and signal amplifier for mechanical stimuli, triggering various biomechanical effects in response to mechanical stress. Inhibiting YAP expression helps maintain vascular homeostasis in endothelial cells [113]. SIRT1 can influence nuclear output and degradation by deacetylating YAP, downregulate the expression of YAP target genes, and inhibit the nucleocytoplasmic shuttling of YAP protein [88]. SIRT1 deacetylates the YAP2 protein, increasing YAP2/TEAD4 binding and activation and promoting cell growth in hepatocellular carcinoma cells. YAP1 expression is also positively correlated with VEGFA expression and secretion, which promotes angiogenesis in renal carcinoma environments [89], 90], 114]. This evidence is extrapolated from atherosclerotic mouse models and cancer cell studies. Direct evidence in the context of stress-induced vascular senescence is lacking.

## 6 Discussion

In summary, SIRT1 can participate in various physiological processes to alleviate stress-induced vascular senescence induced by irritants through multiple effector molecules and signaling pathways, in which “TP53” and “SOD” are among the top 20 molecules involved in oxidative stress (Figure 4), while “IL4,” “VCAM1,” “IL1B,” “IL10,” and other molecules rank among the top 20 in the inflammatory response process (Figure 5), suggesting that the SIRT1 protein can help improve stress-induced vascular senescence through antioxidant and anti-inflammatory effects. Meanwhile, molecules such as “AKT,” “TP53,” “HIF1A,” “MAPK,” “NRF2,” “FOXO1,” and “FOXO3” can interact with SIRT1 (Figure 2), indicating that SIRT1 is also involved in regulating autophagy, cell proliferation, and other processes and that it alleviates stress-induced vascular senescence.

This review offers a novel, network-guided synthesis of SIRT1-mediated regulation in stress-induced vascular senescence, setting it apart from previous reviews of SIRT1 and stress-induced vascular senescence. By integrating STRING/Cytoscape protein-interaction network analysis with a systematic pathway summary focused specifically on stress-driven premature stress-induced vascular senescence, rather than general aging or atherosclerosis, we prioritize mechanistic nodes with the strongest functional links to senescence phenotypes. This work provides an actionable, evidence-weighted framework for interpreting context-dependent SIRT1 signaling and identifying high-priority targets for future intervention in stress-induced vascular senescence.

## 7 Conclusions and future perspectives

### 7.1 Directions for future research

Current research on SIRT1 and stress-induced vascular senescence mainly depends on *in vitro* cell experiments, rodent animal models, and bioinformatic analysis of protein interaction networks, while several research gaps still need to be addressed in follow-up work.

First, most existing studies focus on the regulatory role of SIRT in vascular endothelial cells, whereas the differential regulatory effects of SIRT1 in vascular smooth muscle cells and vascular adventitial cells under persistent stress have not been systematically compared, and the intercellular crosstalk mediated by SIRT1 among multiple vascular cell types requires further clarification.

Second, this review summarizes multiple signaling pathways through which SIRT1 alleviates stress-induced vascular senescence, but the dynamic crosstalk among these pathways under the simultaneous stimulation of oxidative stress, inflammation, and autophagy dysfunction remains unclear. Moreover, the main molecules that coordinate these signaling networks still need to be further identified.

Third, most of the existing relevant evidence is derived from preclinical models, and there is a lack of systematic research on human vascular specimens and long-term population studies to verify whether the activity and expression level of SIRT1 can reflect the severity of stress-induced vascular senescence in clinical populations. Fourth, this study identifies multiple upstream microRNAs that regulate SIRT1, but few studies have examined tissue-targeted intervention strategies to modulate the expression of these non-coding RNAs and further activate SIRT1 to alleviate stress-induced vascular senescence. Fifth, combined interventions targeting multiple drivers of aging, such as RAAS activation, mitochondrial dysfunction, and telomere attrition through the SIRT1 pathway, still lack sufficient preclinical verification.

Thus, future research should focus on exploring compound intervention strategies centered on SIRT1 to simultaneously block multiple pathological processes involved in stress-induced vascular senescence.

## 8 Conclusions

In summary, SIRT1 functions as a key NAD + -dependent deacetylase that regulates stress-induced vascular senescence through multiple interconnected signaling pathways, primarily by counteracting oxidative stress and inflammation. Based on our network analysis and evidence-weighted synthesis, SIRT1/Nrf2, SIRT1/FoxOs, SIRT1/NF-κB, and SIRT1/eNOS/NO appear to be the most robust and relevant pathways underlying stress-induced vascular senescence. Future studies should focus on validating these main network-central nodes in standardized stress-induced endothelial senescence models, comparing SIRT1 signaling across diverse vascular beds and cell types, and linking SIRT1 modulation to clinically recognized indicators of stress-induced vascular senescence such as endothelial function and arterial stiffness. Further investigation is also required to clarify the context-dependent and dose-dependent effects of SIRT1 activation and to validate key pathways in human ex vivo vascular tissue, thus enhancing translational relevance. Overall, this review identifies SIRT1 as a promising target for the prevention and treatment of stress-induced vascular senescence and its associated CVDs.
